# A Case Report of Malignant Struma Ovarii With Papillary Thyroid Carcinoma

**DOI:** 10.1002/ccr3.71603

**Published:** 2025-12-03

**Authors:** Nixon Dangol, Sajiva Aryal, Sharmila Parajuli, Riti Amatya, Suraj Sharma, Ranga Bahadur Basnet

**Affiliations:** ^1^ Kathmandu Model Hospital and Institute of Health Sciences Kathmandu Nepal; ^2^ National Academy of Medical Sciences Bir Hospital Mahaboudha Nepal

**Keywords:** immunohistochemistry, malignant struma ovarii, papillary thyroid cancer, teratoma

## Abstract

Struma ovarii is a monodermal variant of mature teratoma, primarily composed of thyroid tissue. It is a rare ovarian tumor with nonspecific clinical and imaging features. While most cases are benign, a small proportion can undergo malignant transformation, most commonly to papillary carcinoma. Due to its rarity, diagnostic criteria and management guidelines remain poorly defined. A young adult female presented with abdominal pain in our hospital. Imaging studies including ultrasonography and CT scan revealed a midline complex cystosolid mass. The patient underwent right‐sided cystectomy and salpingo‐oophorectomy. Histopathological and immunohistochemical analysis confirmed the diagnosis of malignant struma ovarii. Malignant transformation in struma ovarii is uncommon and follows the same diagnostic criteria as thyroid malignancies. Clinical presentation and imaging features are often nonspecific, and diagnosis relies on histopathology and immunohistochemistry. Surgical resection is the preferred treatment, with the extent determined by tumor characteristics and patient factors. Malignant struma ovarii is a rare ovarian tumor requiring careful histopathological evaluation for diagnosis. Awareness of this entity is crucial for guiding appropriate management.

## Key Clinical Message

- Malignant struma ovarii is a rare malignant condition that currently lacks unanimous standard treatment guidelines.
- This article addresses the most recent approaches which include patient‐centered therapy and monitoring guidelines for the management of malignant struma ovarii.

## 1 Introduction

Struma ovarii is a rare ovarian tumor characterized by the presence of thyroid tissue that makes up at least half of the total ovarian mass [1]. Struma ovarii is most frequently seen in individuals between the ages of 40 and 60 and represents about 3% of ovarian teratomas and 0.3% of all ovarian tumors [2, 3, 4]. Most of the struma are benign mature cystic teratomas [5]. On the other hand, malignant transformation of struma ovarii is extremely rare and occurs in less than 5% of cases [6, 7, 8]. Preoperative diagnosis is difficult because of the lack of distinct clinical and imaging features, resulting in a high rate of misdiagnosis [5, 6, 7]. Consequently, a definitive diagnosis is achieved through histopathological analysis [5]. When metastatic lesions are not present, the diagnosis of malignant struma ovarii is based on criteria used for diagnosing thyroid carcinoma [9]. This includes characteristics like ground glass nuclei, invasion of blood vessels, and mitotic activity [9]. While both papillary and follicular carcinomas have been found in struma ovarii, papillary carcinoma is more frequently seen [3, 10]. There is no unanimous consensus regarding a standard treatment protocol for malignant struma ovarii [9]. Management after the removal of the pelvic mass is tailored to each patient, based on individual circumstances and the medical team's decisions, as suggested by various studies [9]. We report a case of malignant struma ovarii in a young adult patient who presented with abdominal pain and was diagnosed post operatively following laparoscopic salpingo‐oophorectomy.

## 2 Case Presentation

### 2.1 Case History and Examination

A young adult woman presented with a 9‐day history of progressive abdominal pain. Her last menstrual period occurred 13 days prior to the outpatient visit, with regular cycles, average flow, and moderate dysmenorrhea. She denied loss of appetite, significant weight loss, heavy or prolonged menstrual bleeding, dyspareunia, and postcoital bleeding. The patient had no significant past or family history. On examination, she appeared in fair condition with stable vital signs. Her abdomen was soft and non‐tender, without palpable masses. A per speculum examination revealed a normal vulva and vagina, while the cervix was edematous with discharge. Per vaginal examination identified a normal‐sized uterus, right fornix fullness, and a firm, mobile mass measuring approximately 10 cm in the right adnexa.

### 2.2 Investigations

Initial laboratory investigations, including thyroid function tests, were normal, indicating the patient was euthyroid. Abdominal ultrasound revealed a heteroechoic cystosolid mass lesion in the right adnexa measuring about 9.9 × 5.9 cm. A subsequent CT scan of the abdomen and pelvis confirmed a large, heterogeneous cystosolid mass lesion measuring 9.4 × 7.2 cm with lesion bulk in the midline extending to the right side of the pelvis. No ascites or deposits were identified on CT. Tumor markers, including CA‐125, CEA, beta‐hCG, LDH, and AFP, were normal.

### 2.3 Provisional Diagnosis

Based on the initial clinical presentation, examination, and subsequent imaging studies, a diagnosis of malignant right adnexal complex cyst was made.

### 2.4 Treatment

The patient was admitted for monitoring and subsequently underwent laparoscopic staging, which included right‐sided cystectomy, salpingo‐oophorectomy, iliac lymph node sampling, and peritoneal/mesenteric sampling. The procedure revealed minimal peritoneal fluid. The primary finding was a complex right adnexal mass, measuring approximately 10 × 8 cm, originating from the right ovary. The mass exhibited both solid and cystic components and was adherent to a 5 × 1 cm section of the fallopian tube. The cut surface of the mass was predominantly solid, grayish white, with focal areas of necrosis and hemorrhage. The postoperative course was uneventful.

### 2.5 Histopathological Examination

**FIGURE 1:**
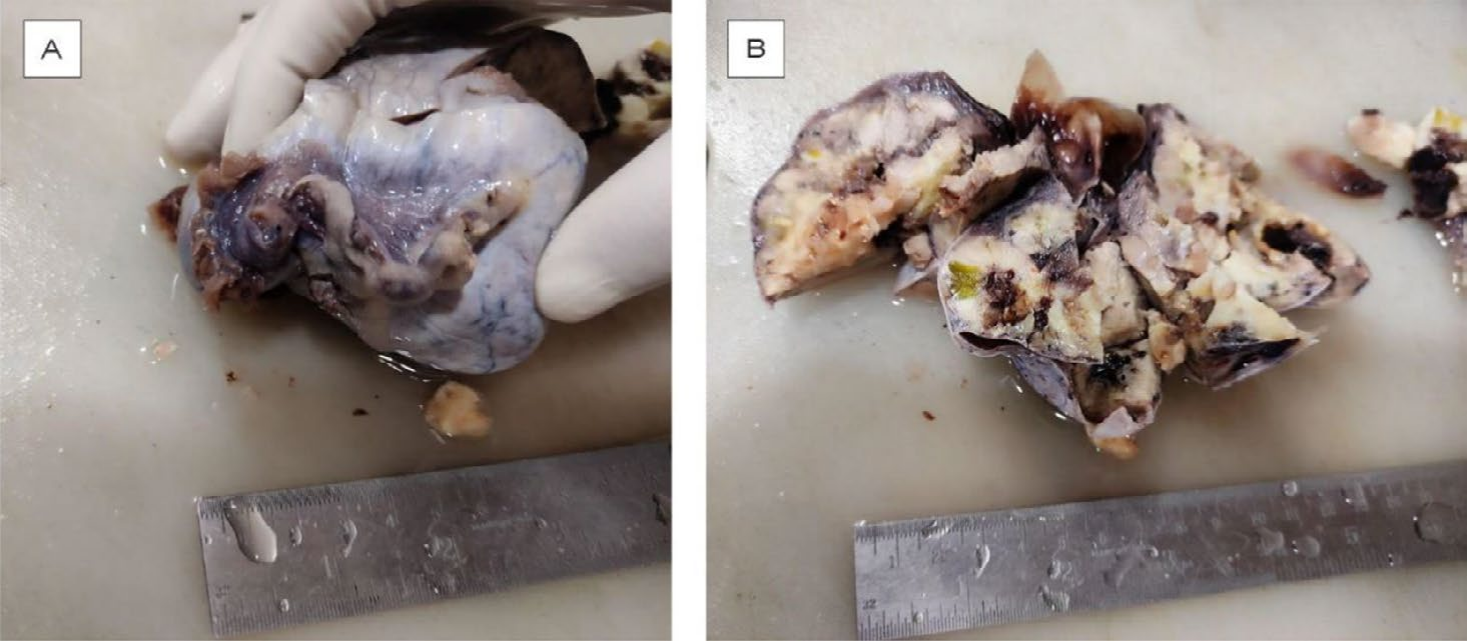
(A) Gross specimen of right adnexal mass measuring 9 × 8 × 6 cm along with adherent fallopian tube. (B) Cut section of right adnexal mass showing multiloculated solid grayish white areas along with areas of necrosis and hemorrhage.

The gross examination of the excised specimen included five containers: (1) a right iliac lymph node, consisting of grayish‐white tissue measuring 2.5 × 0.5 cm; (2) a peritoneal sample from right side of the pelvis, containing five lymph nodes, the largest measuring 0.7 cm in diameter, negative for tumor cells, exhibiting reactive lymphadenitis (lymph node status: 0/5); (3) an omental sample with three pieces of fibrofatty tissue (5 × 3 cm) and no identified lymph nodes or deposits; (4) a right adnexal cystosolid mass, measuring 9 × 8 × 6 cm, adherent to a 5 × 1 cm section of the fallopian tube (Figure 1A), with a predominantly solid grayish‐white cut surface showing areas of necrosis and hemorrhage (Figure 1B); and (5) 10 mL of straw‐colored peritoneal wash fluid.

**FIGURE 2:**
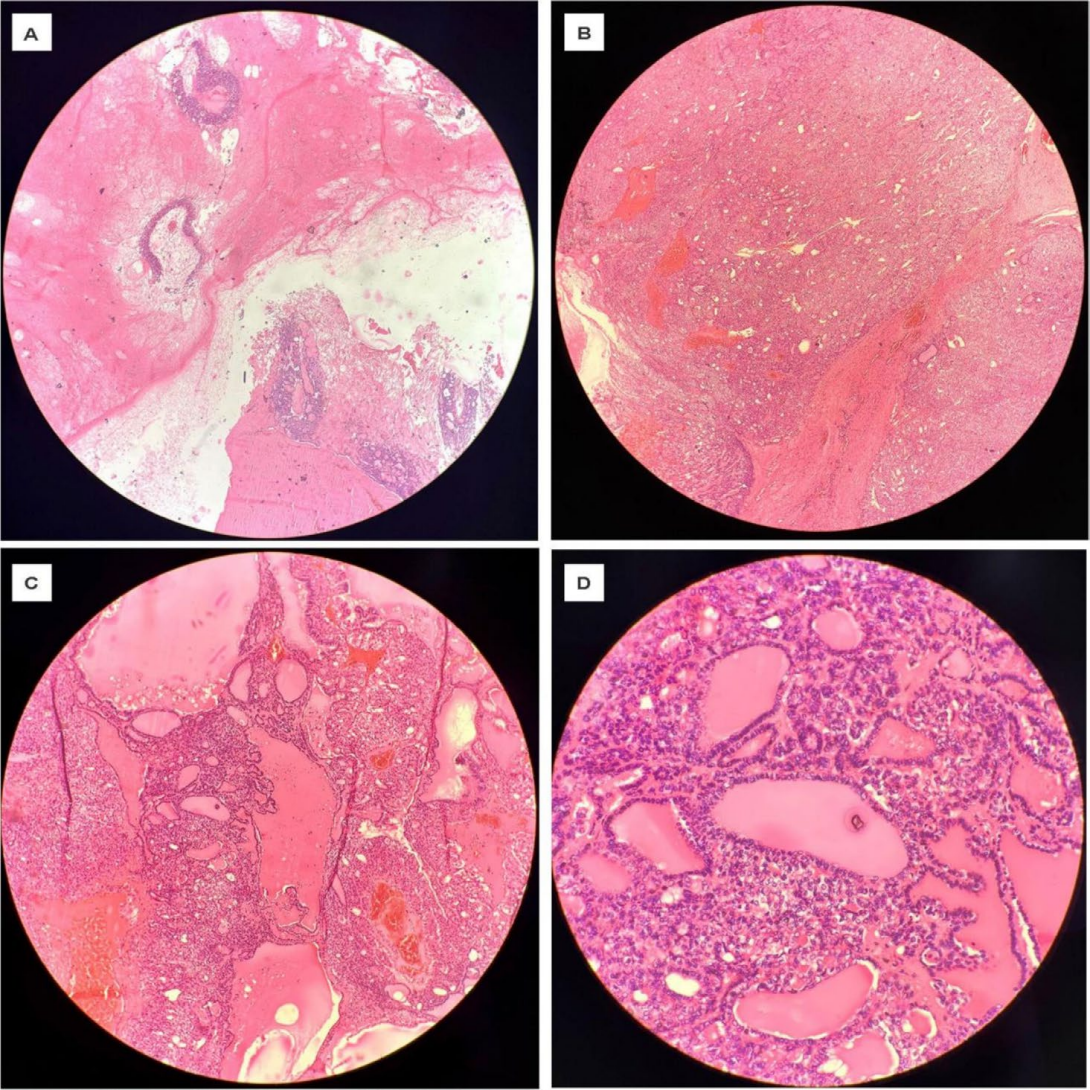
(A) Histopathology from right adnexal cyst showing normal ovary and areas of struma ovary (hematoxylin and eosin stain; 4×). (B) Tumor cells arranged in nests, macro and microfollicles along with papillary pattern having central fibrovascular core (hematoxylin and eosin stain; 4×). (C) Histopathology from right adnexal cyst showing tumor cells are arranged in follicles, macrofollicles and papillary pattern. Areas of normal looking thyroid along with colloid are seen. Areas of necrosis and hemorrhage were also identified (hematoxylin and eosin stain; 10×). (D) Individual tumor cells show mild to moderate pleomorphism, moderate eosinophilic cytoplasm. Nuclear features showed nuclear clearing, overlapping crowding and nuclear grooving (hematoxylin and eosin stain; 40×).

Histologically, the right adnexal mass comprised tumor cells arranged in nests, macro and microfollicles, and a papillary pattern with a central fibrovascular core (Figure 2B). These tumor cells showed mild to moderate pleomorphism, moderate eosinophilic cytoplasm, nuclear clearing, overlapping, crowding, and nuclear grooving (Figure 2D). Areas of necrosis and hemorrhage were identified. Adjacent areas contained normal‐looking thyroid follicles filled with colloid (Figure 2C). No other components of teratoma were identified. The ovarian parenchyma was present (Figure 2A); however, invasion of the ovarian surface or fallopian tube was not observed. The fallopian tube appeared histologically normal and free of tumor cells. Microscopic examination of the right iliac lymph node revealed fibrofatty tissue only with no lymph nodes identified. The peritoneal sample from the right side of the pelvis showed five reactive lymph nodes, all negative for tumor cells. The omental sample comprised fibrofatty tissue, with no tumor cells. Cytology of the peritoneal fluid revealed mesothelial cells with few lymphocytes and macrophages, without atypical or malignant cells.

### 2.6 Immunohistochemistry

**FIGURE 3:**
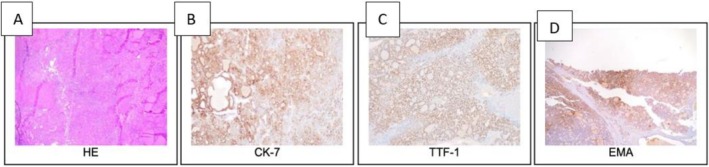
(A) Hematoxylin and eosin‐stained tumor cells. Immunohistochemical analysis showing (B) CK‐7 score 4+, (C) TTF‐1 score 3+, and (D) EMA moderate reactivity score 2+.

Hematoxylin and eosin (H&E) staining was performed on the tumor cells in parallel with immunohistochemistry (IHC) to provide complementary histological and molecular information (Figure 3A). Immunohistochemical analysis showed strong immunoreactivity for Cytokeratin 7 (CK‐7) (score 4+), indicating an epithelial tumor likely of ovarian and thyroid origin (Figure 3B). PAX8, a key marker for thyroid and Mullerian tissues, also demonstrated strong nuclear reactivity (score 4+), supporting the diagnosis of struma ovarii. Thyroid transcription factor‐1 (TTF‐1) was strongly positive (score 3+), reinforcing the presence of thyroid‐originating carcinoma in the ovarian mass (Figure 3C). Ki‐67 showed moderate proliferative activity (10%–12%), indicating low potential tumor growth. Epithelial membrane antigen (EMA) displayed moderate reactivity (score 2+), confirming epithelial differentiation (Figure 3D). BRAF V600E showed moderate reactivity (score 2+) in papillary areas, suggesting malignant thyroid components. Additionally, HBME1 showed patchy reactivity in the papillary regions, further supporting the presence of papillary thyroid carcinoma and aiding in distinguishing it from other tumor types.

### 2.7 Outcome and Follow‐Up

In conclusion, the diagnosis of malignant struma ovarii with papillary thyroid carcinoma arising from the right ovary was confirmed. The tumor was confined to the ovary with an intact capsule, and there was no involvement of the fallopian tube or ovarian surface. Lymphovascular invasion, lymph node metastasis, or peritoneal/omental involvement was not observed. The final pathological staging was T1_a_N_0_M_x_, corresponding to Stage IA. The patient was scheduled for a follow‐up visit in the gynecology outpatient department (OPD) and was counseled regarding further treatment. This included a discussion on the risk of recurrence and the importance of close monitoring of thyroglobulin levels. Post‐operative thyroid ultrasound was normal. Post‐operative levels of serum thyroglobulin antibodies and serum thyroid peroxidase antibodies were found to be within normal limits. Although whole‐body PET‐CT or MRI can be considered in cases with suspected metastatic or extra‐ovarian involvement, these were not indicated in our patient given the absence of clinical and biochemical suspicion.

## 3 Discussion

Malignant struma ovarii presents significant diagnostic and therapeutic challenges due to its rarity and diverse clinical features [6, 11, 12]. Malignant struma ovarii typically presents as a unilateral adnexal mass in patients during their fifth or sixth decade of life [2, 3, 13]. However, the patient presented in our case was a young adult female in her late twenties. These tumors are often large, and most patients are euthyroid, with only about 2.78% experiencing hyperthyroidism, while it varies from 5% to 15% in older studies [12, 14, 15]. In our case, the thyroid function profile was within normal limits. The symptoms are often nonspecific, such as pelvic pain, making diagnosis clinically challenging [16, 17]. In our case, the patient experienced vague abdominal pain without any other notable symptoms, highlighting the diagnostic difficulties commonly associated with this condition.

Histological examination is essential for diagnosing malignant struma ovarii [8, 14, 18]. These tumors typically contain both benign and malignant thyroid tissue, with key indicators of malignancy including capsular and vascular invasion, cellular atypia, and mitotic activity [4, 14, 19]. The Pardo–Mindan criteria highlight that nuclear changes alone are not enough for diagnosis and require additional evidence of capsular or peritoneal invasion [7, 20]. Histopathological examination in our case did not show evidence of capsular or peritoneal invasion, but a high degree of nuclear atypia was noted. However, these criteria are still a subject of debate, and obtaining sufficient tissue samples is crucial to ensure an accurate diagnosis.

Ultrasonography is the first‐line imaging method for evaluating ovarian masses, particularly in adolescents, as it provides essential details about the size, volume, and morphology of a cyst, including the presence of solid components and vascularity via Doppler examination [21, 22]. Transvaginal sonography (TVS) is the preferred technique for assessing adnexal masses in general, with the Ovarian‐Adnexal Reporting and Data System (O‐RADS) offering a risk classification system for malignancy based on ultrasound findings [22]. A meta‐analysis by Vara et al. found that O‐RADS has high sensitivity (97%) and moderate specificity (77%), making it a valuable tool for preoperative malignancy assessment [22]. Contrast‐enhanced CT is also commonly used to assess disease spread and detect features suggestive of malignancy, such as thick septa, solid areas, and papillary projections [14, 21, 22]. However, preoperative imaging remains nonspecific, and histopathological analysis remains the gold standard for definitive diagnosis [14, 22].

Leuștean et al. suggest that immunohistochemistry (IHC) could be particularly helpful in doubtful cases, complementing traditional morphology‐based methods like the Pardo Mindán criteria for diagnosing malignant struma ovarii (MSO) [14]. By using thyroid‐specific markers such as thyroglobulin and TTF‐1, IHC helps confirm the presence of thyroid tissue within ovarian tumors, distinguishing MSO from other ovarian neoplasms [12, 23, 24]. Advanced markers like CK19, galectin‐3, and CD56 further enhance the sensitivity and specificity in differentiating malignant from benign thyroid tissue, with combined detection achieving increased diagnostic accuracy [12, 14, 24]. For rare scenarios where MSO mimics other tumors, such as strumal carcinoid or medullary thyroid carcinoma, IHC markers such as calcitonin or neuroendocrine markers are indispensable for accurate classification [10, 12, 24]. Genetic testing, particularly the detection of BRAF mutations (V600E, K601E, and TV599‐600M alterations), has emerged as a more accurate method for distinguishing malignant from benign cases [17, 23, 25]. BRAF mutations are strongly linked to malignant transformation, whereas NRAS mutations are more commonly associated with the follicular variant of papillary thyroid cancer [12, 17, 25]. Additionally, molecular studies suggest that the pathogenesis of MSO shares similarities with thyroid cancer [12, 23, 25]. Ayhan et al. mentioned a study conducted by Tan et al. that molecular sequencing such as for BRAF mutations could further categorize MSO and guide treatment, as MSO patients with BRAF mutations tend to have worse clinical outcomes [26]. However, Bellini et al. in their review found no significant correlation between BRAF mutation status and prognosis [27].

Treatment strategies are tailored based on the patient's age, desire for fertility, and disease stage [8, 23, 28]. In a review of 170 cases of MSO confined to the ovary, 4 out of 34 (11.8%) recurrent patients and 4 out of 136 (2.9%) non‐recurrent patients received chemotherapy [8]. However, the effect of chemotherapy on disease‐free survival (DFS) in stage I MSO was not statistically significant [8]. In a study by DeSimone et al., a patient with mixed malignant struma ovarii received courses of phenylalanine mustard, Adriamycin, and vincristine but experienced disease progression [6].

In patients with MSO and overt hyperthyroidism, surgery should be postponed until adequate control is achieved (typically 3–8 weeks) using beta blockers and thionamides, with iodine if urgent control is required [15, 29].

For younger patients, fertility‐preserving surgery is preferred as in our case, while more radical procedures, such as total abdominal hysterectomy with bilateral salpingo‐oophorectomy followed by total thyroidectomy and radioactive iodine (^131^I) therapy are recommended for postmenopausal women or those with advanced disease (capsular/peritoneal invasion) [14, 26]. However, some cases advocate for a more aggressive approach regardless of age or initial stage, contrasting with the fertility‐sparing preference observed in other recent literature [14, 24, 26]. A study analyzing 164 cases of MSO confined to the ovary found that patients who underwent bilateral or unilateral salpingo‐oophorectomy had better disease‐free survival (DFS) than those who underwent ovarian cystectomy (*p* < 0.001) [8]. Multivariate Cox regression analysis confirmed adnexal surgery (USO or BSO) as an independent factor influencing DFS, with a significantly lower risk of recurrence compared to ovarian cystectomy [8]. However Cox regression analysis on recurrence‐free survival (RFS) and overall survival conducted by Li et al. indicates that while univariate analysis showed some associations, further Cox proportional hazard analyses failed to identify any factors with statistical significance for RFS [23].

Aggressive therapy is followed by serial monitoring of serum thyroglobulin levels and frequent whole body ^131^I scans as elevated serum thyroglobulin levels and iodine uptake are key markers for monitoring tumor recurrence [6, 14, 30, 31].

This aggressive approach is needed in advanced cases of MSO as the risk of recurrence rate is high at 22%–35% following therapy [8, 14]. However, the risk of recurrence rate in localized cases of MSO is 7.5% following conservative therapy [23]. On the other hand, patients with benign features following conservative therapy are treated with Levothyroxine to suppress TSH levels, followed by long‐term follow‐up as definitive (radical) surgery was associated with better progression‐free survival (81.4%) compared to conservative surgery (63.3%) [11, 26]. Additionally, near‐total thyroidectomy followed by radioiodine ablation is recommended when thyroglobulin levels exceed the baseline during follow‐up after conservative therapy, aiding in the detection and treatment of recurrent disease [11]. But no official unanimous consensus has been established to follow a standard treatment protocol. In our patient, we deferred thyroidectomy in favor of risk‐adapted management.

Several studies highlight the importance of evidence‐based recommendations for managing MSO, particularly following American Thyroid Association (ATA) guidelines [23, 27, 32]. Based on the recent ATA guidelines, suppressed TSH goals for differentiated thyroid cancers are determined based on dynamic risk stratification criteria, which is between 0.1 and 0.5 mIU/L for indeterminate response, and 0.5 and 2.0 mIU/L for excellent response [11, 33]. However, no such specific risk‐stratified targets for suppressed TSH exist for MSO. Suppressed TSH goals for our patient were also aimed between 0.5 and 2 mIU/L, owing to her low risk disease.

Ayhan et al. further suggest stratifying patients based on risk, which is modeled after ATA guidelines, categorizing low‐risk cases as tumors confined within struma ovarii, measuring < 2 cm, and exhibiting well‐differentiated histology [26]. These cases may be managed with oophorectomy, levothyroxine suppression to reduce the potential stimulatory effect of TSH on residual or recurrent disease, and thyroglobulin monitoring [26]. High‐risk cases (extraovarian spread, large tumors, or synchronous thyroid cancer) generally warrant thyroidectomy and radioiodine therapy with TSH suppression [26]. For equivocal presentations, thyroid ultrasound and fine needle aspiration of synchronous thyroid lesions and molecular testing of the ovarian tumor may be considered. Ayhan et al. also conducted univariate analysis and identified several poor progression‐free survival (PFS) factors, including younger age (< 43 years), whole stromal cysts, tumor diameter > 95 mm, non‐papillary thyroid cancer histology, and lymphovascular invasion [26].

Bellini references Egan et al.'s findings that high‐risk stratification does not always align with the use of radioactive iodine (RAI) therapy as per ATA guidelines [27]. TERT promoter mutations are found to be linked with RAI‐resistant cases of MSO, with a higher likelihood of metastasis [34]. Unlike classical thyroid cancer, RAI therapy in malignant struma ovarii (MSO) is not consistently applied, even in high‐risk cases [27, 32]. However, Ayhan et al. demonstrated that administering RAI before treatment failure significantly improved PFS (94.5% vs. 64.8%, *p* = 0.041) [26].

In our case, our patient fell into the poor PFS category and was thoroughly counseled about their prognosis and is scheduled for next follow up visit.

## 4 Conclusion

Malignant struma ovarii is a rare and complex condition that demands careful evaluation and personalized management. Despite the challenges it presents, most cases have an excellent prognosis with evidence‐based tailored treatment. Ongoing research is essential to refine diagnostic criteria and develop standardized treatment protocols for these uncommon tumors.

## Author Contributions

**Nixon Dangol:** methodology, supervision, writing – original draft, writing – review and editing. **Sajiva Aryal:** writing – original draft, writing – review and editing. **Sharmila Parajuli:** supervision, writing – review and editing. **Riti Amatya:** supervision, writing – review and editing. **Suraj Sharma:** writing – original draft, writing – review and editing. **Ranga Bahadur Basnet:** writing – review and editing.

## Funding

The authors have nothing to report.

## Ethics Statement

Ethics approval was not required for this case report, as it is exempt under the policy set by the Institutional Review Committee of Public Health Concern Trust (PHECT), Nepal, for Kathmandu Model Hospital. An exemption form was issued to conduct the study and can be provided upon request.

## Consent

Written informed consent was obtained from the patient for publication of this case report and any accompanying images. A copy of the written consent is available for review by the Editor‐in‐Chief of this journal upon request.

## Conflicts of Interest

The authors declare no conflicts of interest.

## Data Availability

The data that support the findings of this study are available on request from the corresponding author. The data are not publicly available due to privacy or ethical restrictions.
